# The cross-sectional GRAS sample: *A comprehensive phenotypical data collection of schizophrenic patients*

**DOI:** 10.1186/1471-244X-10-91

**Published:** 2010-11-10

**Authors:** Katja Ribbe, Heidi Friedrichs, Martin Begemann, Sabrina Grube, Sergi Papiol, Anne Kästner, Martin F Gerchen, Verena Ackermann, Asieh Tarami, Annika Treitz, Marlene Flögel, Lothar Adler, Josef B Aldenhoff, Marianne Becker-Emner, Thomas Becker, Adelheid Czernik, Matthias Dose, Here Folkerts, Roland Freese, Rolf Günther, Sabine Herpertz, Dirk Hesse, Gunther Kruse, Heinrich Kunze, Michael Franz, Frank Löhrer, Wolfgang Maier, Andreas Mielke, Rüdiger Müller-Isberner, Cornelia Oestereich, Frank-Gerald Pajonk, Thomas Pollmächer, Udo Schneider, Hans-Joachim Schwarz, Birgit Kröner-Herwig, Ursula Havemann-Reinecke, Jens Frahm, Walter Stühmer, Peter Falkai, Nils Brose, Klaus-Armin Nave, Hannelore Ehrenreich

**Affiliations:** 1 Division of Clinical Neuroscience, Max Planck Institute of Experimental Medicine, Göttingen, Germany; 2 Department of Psychiatry and Psychotherapy, Ecumenical Hospital Hainich, Germany; 3 Hospital of Psychiatry and Psychotherapy, Center for Integrative Psychiatry, Kiel, Germany; 4 Karl-Jaspers-Hospital, Psychiatric Federation Oldenburger Land, Bad Zwischenahn, Germany; 5 Department of Psychiatry II, Ulm University, District Hospital Günzburg, Germany; 6 Department of Psychiatry and Psychotherapy, Hospital Fulda, Germany; 7 Department of Psychiatry and Psychotherapy, Isar-Amper-Hospital, Taufkirchen (Vils), Germany; 8 Department of Psychiatry and Psychotherapy, Reinhard-Nieter Hospital, Wilhelmshaven, Germany; 9 Vitos Hospital of Forensic Psychiatry Eltville, Eltville, Germany; 10 Vitos Hospital of Psychiatry and Psychotherapy Merxhausen, Kassel, Germany; 11 Department of Psychiatry and Psychotherapy, University of Rostock, Germany; 12 Hospital of Forensic Psychiatry, Moringen, Germany; 13 Hospital of Psychiatry and Psychotherapy Langenhagen, Regional Hospitals Hannover, Germany; 14 Vitos Hospital of Psychiatry and Psychotherapy, Bad Emstal-Merxhausen, Germany; 15 Addiction Hospital "Am Waldsee", Rieden, Germany; 16 Department of Psychiatry and Psychotherapy, University Medical Center of Bonn, Germany; 17 Vitos Hospital of Psychiatry and Psychotherapy Merxhausen, Hofgeismar, Germany; 18 Vitos Haina Forensic Psychiatric Hospital, Haina, Germany; 19 Department of Psychiatry and Psychotherapy, Regional Hospitals Hannover, Wunstorf, Germany; 20 Dr. K. Fontheim's Hospital for Mental Health, Liebenburg, Germany; 21 Department of Psychiatry and Psychotherapy, Hospital Ingolstadt, Germany; 22 Department of Psychiatry and Psychotherapy, Hospital Lübbecke, Germany; 23 Hospital of Psychiatry and Psychotherapy, Rickling, Germany; 24 Georg-Elias-Müller-Institute for Psychology, University of Göttingen, Germany; 25 Department of Psychiatry and Psychotherapy, University Medical Center of Göttingen, Germany; 26 Biomedical NMR Research GmbH, Max Planck Institute of Biophysical Chemistry, Göttingen, Germany; 27 Department of Molecular Biology of Neuronal Signals, Max Planck Institute of Experimental Medicine, Göttingen, Germany; 28 Department of Molecular Neurobiology, Max Planck Institute of Experimental Medicine, Göttingen, Germany; 29 Department of Neurogenetics, Max Planck Institute of Experimental Medicine, Göttingen, Germany; 30 DFG Research Center for Molecular Physiology of the Brain (CMPB), Germany; 31 Founders of the GRAS Initiative

## Abstract

**Background:**

Schizophrenia is the collective term for an exclusively clinically diagnosed, heterogeneous group of mental disorders with still obscure biological roots. Based on the assumption that valuable information about relevant genetic and environmental disease mechanisms can be obtained by association studies on patient cohorts of ≥ 1000 patients, if performed on detailed clinical datasets and quantifiable biological readouts, we generated a new schizophrenia data base, the GRAS (Göttingen Research Association for Schizophrenia) data collection. GRAS is the necessary ground to study genetic causes of the schizophrenic phenotype in a 'phenotype-based genetic association study' (PGAS). This approach is different from and complementary to the genome-wide association studies (GWAS) on schizophrenia.

**Methods:**

For this purpose, 1085 patients were recruited between 2005 and 2010 by an invariable team of traveling investigators in a cross-sectional field study that comprised 23 German psychiatric hospitals. Additionally, chart records and discharge letters of all patients were collected.

**Results:**

The corresponding dataset extracted and presented in form of an overview here, comprises biographic information, disease history, medication including side effects, and results of comprehensive cross-sectional psychopathological, neuropsychological, and neurological examinations. With >3000 data points per schizophrenic subject, this data base of living patients, who are also accessible for follow-up studies, provides a wide-ranging and standardized phenotype characterization of as yet unprecedented detail.

**Conclusions:**

The GRAS data base will serve as prerequisite for PGAS, a novel approach to better understanding 'the schizophrenias' through exploring the contribution of genetic variation to the schizophrenic phenotypes.

## Background

Schizophrenia is a devastating brain disease that affects approximately 1% of the population across cultures [1]. The diagnosis of schizophrenia or - perhaps more correctly - of 'the schizophrenias' is still purely clinical, requiring the coincident presence of symptoms as listed in the leading classification systems, DSM-IV and ICD-10 [2,3].

Notably, one of the core symptoms of schizophrenia, namely cognitive deficits, from mild impairments to full-blown dementia, has not yet been considered in these classifications. Biologically, schizophrenia is a 'mixed bag' of diseases that undoubtedly have a strong genetic root. Family studies exploring relative risk of schizophrenia have led to estimates of heritability of about 64-88% [4,5]. Monozygotic twin studies showing concordance rates of 41-65% [6,7] indicate a considerable amount of non-genetic causes, in the following referred to as 'environmental factors'. Already in the middle of the twentieth century, schizophrenia was seen as a 'polygenetic' disease [8] and, indeed, in numerous genetic studies since, ranging from segregation or linkage analyses, genome scans and large association studies, no major 'schizophrenia gene' has been identified [9]. Even recent genome-wide association studies (GWAS) on schizophrenia confirm that several distinct loci are associated with the disease. These studies concentrated on endpoint diagnosis and found odds ratios for single markers in different genomic regions ranging from 0.68 to 6.01 [10], essentially underlining the fact that - across ethnicities - in most cases these genotypes do not contribute more to the disease than a slightly increased probability.

**Figure 1:**
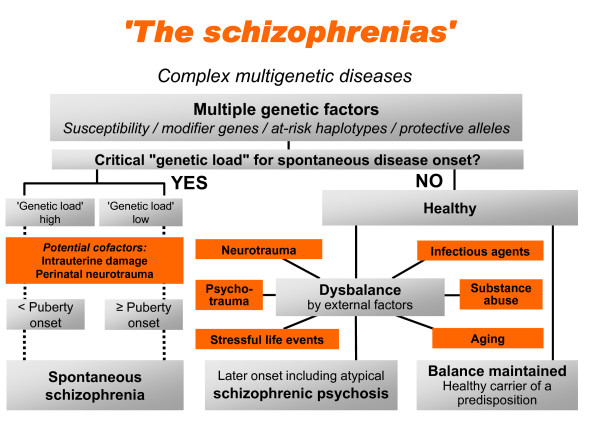
**Schizophrenia is a complex multigenetic disease**. Schizophrenia may be seen as the result of a multifaceted interplay between multiple causative factors, including several genetic markers and a variety of different environmental risks. Cases with a critical genetic load may not need additional external/environmental co-factors, whilst in others, the interaction of a certain genetic predisposition with environmental co-factors is required for disease onset (modified from [84]).

We hypothesize that an interplay of multiple causative factors, perhaps thousands of potential combinations of genes/genetic markers and an array of different environmental risks, leads to the development of 'the schizophrenias', as schematically illustrated in Figure 1. There may be cases with a critical genetic load already present without need of additional external co-factors, however, in most individuals, an interaction of a certain genetic predisposition with environmental co-factors is apparently required for disease onset. In fact, not too much of an overlap may exist between genetic risk factors from one schizophrenic patient to an unrelated other schizophrenic individual, explaining why it is basically impossible to find common risk genes of schizophrenia with appreciable odds ratios. One GRAS working hypothesis is that in the overwhelming majority of cases, schizophrenia is the result of a 'combination of unfortunate genotypes'.

If along the lines of traditional human genetics all attempts to define schizophrenia as a 'classical' genetic disease have largely failed, how can we learn more about the contribution of genes/genotypes to the disease phenotype? Rather than searching by GWAS for yet other schizophrenia risk genes, we designed an alternative and widely complementary approach, termed PGAS (phenotype-based genetic association study), in order to explore the contribution of certain genes/genetic markers to the schizophrenic phenotype. To launch PGAS, we had to establish a comprehensive phenotypical data base of schizophrenic patients, the GRAS (Göttingen Research Association for Schizophrenia) data collection. Very recently, we have been able to demonstrate proof-of-concept for the PGAS approach [[11], and Grube et al: Calcium-activated potassium channels as regulators of cognitive performance in schizophrenia, submitted].

Large data bases of schizophrenic patients have been instigated for decades to perform linkage/family studies, treatment trials, genetic or epidemiological studies applying either a cross-sectional or a longitudinal design (e.g. [12-20]). However, for the above introduced PGAS approach, another type of data base is required, and only few of the existing data banks are suited for phenotypical analyses. An example is the 'Clinical Antipsychotic Trial of Intervention Effectiveness (CATIE)', originally set up as a treatment study comparing a first generation antipsychotic drug with several second generation antipsychotics in a multisite randomized double-blind trial [17,21]. The huge amount of data accumulated in the frame of this trial serves now also for GWAS and genotype-phenotype association studies [22-25]. Disadvantages may be that the CATIE data were collected by different examiners in 57 US sites and that comprehensive data for phenotypical analyses are only available for subsamples of the originally included 1493 patients. Another example of a large data base with considerable phenotypical power is the 'Australian Schizophrenia Research Bank (ASRB)' [26]. ASRB operates to collect, store and distribute linked clinical, cognitive, neuroimaging and genetic data from a large sample of patients with schizophrenia (at present nearly 500) and healthy controls (almost 300) [27,28]).

The present paper has been designed (1) to introduce the GRAS data collection, set up as prerequisite and platform for PGAS; (2) to exemplify on some selected areas of interest the potential of phenotypical readouts derived from the GRAS data collection and their internal consistency; (3) to provide a first panel of epidemiological data as a 'side harvest' of this data base; and (4) to enable interested researchers worldwide to initiate scientific collaborations based on this data base.

## Methods

### Ethics

**Figure 2:**
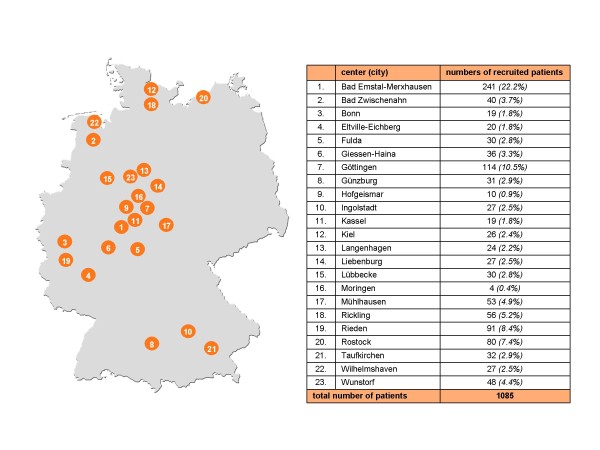
**Collaborating centers and patient numbers**. Map of Germany displaying the locations of all 23 collaborating centers that were visited by an invariable team of traveling investigators. The table next to the map provides numbers of patients examined in each center. Some centers were visited more than once.

The GRAS data collection has been approved by the ethical committee of the Georg-August-University of Göttingen (master committee) as well as by the respective local regulatories/ethical committees of all collaborating centers (Table 1). The distribution of the centers over Germany together with information on the numbers of recruited patients per center is presented in Figure 2.

**Table 1:** GRAS data collection manual: Table of contents

**category**	**content**	**reference in the paper**
		
legal documents/ethical requirements	patient information, informed consent form, confidentiality form, and others...	
patient history	general information (age, sex, ethnicity,...)	→ table 2
	education/employment	→ table 2
	living situation	→ table 2
	legal history	
	medication including side effects	→ table 4
	medical history	
	family history	
	global quality of life^a^	→ table 2 and figure 6
	birth history/traumatic brain injury	
	stressful life events	
	suicidal thoughts/suicide attempts	
	hospitalization history	→ table 2 and figure 6
clinical interviews/ratings	parts of SCID-I: addiction, anxiety, affective disorders, psychotic disorders*^b^	
	Positive and Negative Syndrome Scale* (PANSS)^c^	→ table 2 and figure 6
	Clinical Global Impression* (CGI)^d^	→ table 2 and figure 6
	Global Assessment of Functioning* (GAF)^e^	→ table 2 and figure 6
questionnaires	State-Trait-Anxiety-Inventory* (STAI)^f^	→ table 2 and figure 6
	Brief Symptom Inventory* (BSI)^g^	→ table 2 and figure 6
	Toronto Alexithymia Scale* (TAS)^h^	→ table 2
cognitive tests	premorbid IQ (MWT-B)^i, j^	→ table 3 and figure 7
	reasoning (LPS-3)^k^	→ table 3 and figure 7
	letter-number-span (BZT)^l^	→ table 3 and figure 7
	finger dotting and tapping^m^	→ table 3 and figure 7
	trail making tests (TMT-A and TMT-B)^n^	→ table 3 and figure 7
	verbal fluency (DT/RWT)^o, p^	
	digit-symbol test (ZST)^q^	→ table 3 and figure 7
	verbal memory* (VLMT)^r^	→ table 3 and figure 7
physical examination	Testbatterie zur Aufmerksamkeitsprüfung (TAP)^s^	→ table 3 and figure 7
	general physical examination	
	Cambridge Neurological Inventory (CNI)^t^	→ table 5 and figure 8
	Contralateral Co-Movement Test (COMO)^u^	
	Barnes Akathisia Rating Scale (BARS)^v^	→ figure 8
	Simpson-Angus Scale (SAS)^w^	→ figure 8
	Tardive Dyskinesia Rating Scale (TDRS)^x^	→ figure 8
	Abnormal Involuntary Movement Scale (AIMS)^y^	→ figure 8
	odor testing (ORNI Test)^z^	
	blood sampling (DNA, serum)	

*questionnaires and cognitive tests in respective German versions

^a ^Based on a visual analogue scale (Krampe H, Bartels C, Victorson D, Enders CK, Beaumont J, Cella D, Ehrenreich H: The influence of personality factors on disease progression and health-related quality of life in people with ALS. Amyotroph Lateral Scler 2008, 9:99-107). ^b^Wittchen H-U, Zaudig, M. and Fydrich, T.: SKID-I (Strukturiertes Klinisches Interview für DSM-IV; Achse I: Psychische Störungen). Göttingen: Hogrefe; 1997. ^c^Kay SR, Fiszbein A, Opler LA: The positive and negative syndrome scale (PANSS) for schizophrenia. Schizophr Bull 1987, 13(2):261-276. ^d^Guy W: Clinical Global Impression (CGI). In ECDEU Assessment manual for psychopharmacology, revised National Institue of Mental Health. Rockville, MD; 1976. ^e^AmericanPsychiatricAssociation: Diagnostic and statistical manual of mental disorders, 4th edition (DSM-IV). Washington, DC: American Psychiatric Press; 1994. ^f^Laux L, Glanzmann P, Schaffner P, Spielberger CD: Das State-Trait-Angstinventar (STAI). Weinheim: Beltz; 1981. ^g^Franke GH: Brief Symptom Inventory (BSI). Goettingen: Beltz; 2000. ^h^Kupfer J, Brosig B, Braehler E: Toronto Alexithymie-Skala-26 (TAS-26). Goettingen: Hogrefe; 2001. ^i^Lehrl S, Triebig G, Fischer B: Multiple choice vocabulary test MWT as a valid and short test to estimate premorbid intelligence. Acta Neurol Scand 1995, 91(5):335-345. ^j^Lehrl S: Mehrfach-Wortschatz-Intelligenztest MWT-B. Balingen: Spitta Verlag; 1999. ^k^Horn W: Leistungsprüfsystem (LPS). 2 edition. Goettingen: Hogrefe; 1983. ^l^Gold JM, Carpenter C, Randolph C, Goldberg TE, Weinberger DR: Auditory working memory and Wisconsin Card Sorting Test performance in schizophrenia. Arch Gen Psychiatry 1997, 54(2):159-165. ^m^Chapman RL: The MacQuarrie test for mechanical ability. Psychometrika 1948, 13(3):175-179. ^n^War-Department: Army Individual Test Battery. Manual of directions and scoring. Washington, D.C.: War Department, Adjutant General's Office; 1944. ^o^Kessler J, Denzler P, Markowitsch HJ: Demenz-Test (DT). Göttingen: Hogrefe; 1999. ^p^Aschenbrenner S, Tucha O, Lange KW: Der Regensburger Wortflüssigkeits-Test (RWT). Göttingen: Hogrefe; 2000. ^q^Tewes U: Hamburg-Wechsler Intelligenztest fuer Erwachsene (HAWIE-R). Bern: Huber; 1991. ^r^Helmstaedter C, Lendt M, Lux S: Verbaler Lern- und Merkfåhigkeitstest (VLMT). Goettingen: Beltz; 2001. ^s^Zimmermann P, Fimm B: Testbatterie zur Aufmerksamkeitsprüfung (TAP). Version 1.02c. Herzogenrath: PSYTEST; 1993. ^t^Chen EY, Shapleske J, Luque R, McKenna PJ, Hodges JR, Calloway SP, Hymas NF, Dening TR, Berrios GE: The Cambridge Neurological Inventory: a clinical instrument for assessment of soft neurological signs in psychiatric patients. Psychiatry Res 1995, 56(2):183-204. ^u^Bartels C, Mertens N, Hofer S, Merboldt KD, Dietrich J, Frahm J, Ehrenreich H: Callosal dysfunction in amyotrophic lateral sclerosis correlates with diffusion tensor imaging of the central motor system. Neuromuscul Disord 2008, 18(5):398-407. ^v^Barnes TR: The Barnes Akathisia Rating Scale - revisited. J Psychopharmacol 2003, 17(4):365-370. ^w^Simpson GM, Angus JW: A rating scale for extrapyramidal side effects. Acta Psychiatr Scand Suppl 1970, 212:11-19. ^x^Simpson GM, Lee JH, Zoubok B, Gardos G: A rating scale for tardive dyskinesia. Psychopharmacology (Berl) 1979, 64(2):171-179. ^y^Guy W: Abnormal involuntary movement scale (AIMS). In ECDEU Assessment manual for psychopharmacology, revised National Institute of Mental Health. Rockville, MD; 1976. ^z^ORNI Test (Odor Recognition, Naming and Interpretation Test; developed for the purpose of odor testing in schizophrenics; manuscript in preparation)

### GRAS patients

**Figure 3:**
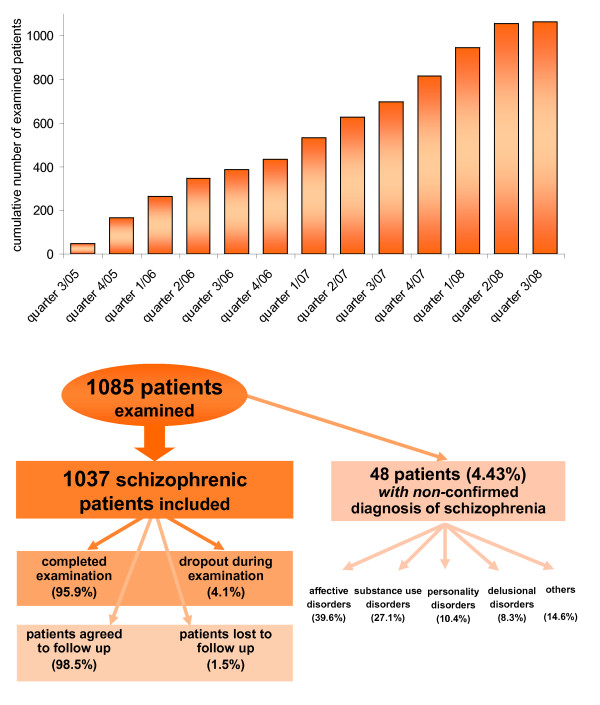
**Patient recruitment and flow:** (a) Recruitment efficiency 2005 - 2008. Cumulative numbers of recruited patients per quarter of the year are shown in bar graphs. Note that steady-state recruitment is ongoing. (b) Patient flow. Of 1085 patients examined, the diagnosis of schizophrenia or schizoaffective disorder could not be confirmed for 48. Instead, alternative diagnoses had to be given.

From September 2005 to July 2008, a total of 1071 patients were examined by the GRAS team of traveling investigators after giving written informed consent, own and/or authorized legal representatives. Since then, low-rate steady state recruitment has been ongoing, among others to build up a new cohort for replicate analyses of genotype-phenotype associations. As of July 2010, 1085 patients have been entered into the data base. They were examined in different settings: 348 (32.1%) as outpatients, 474 (43.7%) as inpatients in psychiatric hospitals, 189 (17.4%) as residents in sheltered homes, 54 (5%) as patients in specific forensic units, and 20 (1.8%) as day clinic patients. Inclusion criteria were (1) confirmed or suspected diagnosis of schizophrenia or schizoaffective disorder according to DSM-IV and (2) at least some ability to cooperate. Recruitment efficiency over the core travel/field study time from 2005 to 2008 and patient flow are shown in Figures 3a and 3b. Of the 1085 patients entered into the data base, a total of 1037 fulfilled the diagnosis of schizophrenia or schizoaffective disorder. For 48 patients the diagnosis of schizophrenia could not be ultimately confirmed upon careful re-check and follow-up. Of the schizophrenic patients, 96% completed the GRAS assessment whereas about 4% dropped out during the examination. Almost all patients agreed to be re-contacted for potential follow-up studies, only 1.5% were either lost to follow-up (present address unknown or deceased) or did not give consent to be contacted again.

### Healthy control subjects

**(1) **For genetic analyses, control subjects, who gave written informed consent, were voluntary blood donors, recruited by the Department of Transfusion Medicine at the Georg-August-University of Göttingen according to national guidelines for blood donation. As such, they widely fulfill health criteria, ensured by a broad pre-donation screening process containing standardized questionnaires, interviews, hemoglobin, blood pressure, pulse, and body temperature determinations. Of the total of 2265 subjects, 57.5% are male (n = 1303) and 42.5% female (n = 962). The average age is 33.8 ± 12.2 years, with a range from 18 to 69 years. Participation as healthy controls for the GRAS sample was anonymous, with information restricted to age, gender, blood donor health state and ethnicity. Comparable to the patient population (Table 2), almost all control subjects were of European Caucasian descent (Caucasian 97.8%; other ethnicities 2%; unknown 0.2%). **(2) **For selected cognitive measures and olfactory testing, 103 additional healthy volunteers were recruited as control subjects (matched with respect to age, gender, and smoking habits). These healthy controls include 67.0% male (n = 69) and 33.0% (n = 34) female subjects with an average age of 39.02 ± 13.87 years, ranging from 18 to 71 years.

**Table 2:** GRAS sample description

		total				men				women				statistics	
					
		N	*%*	mean (sd)	median	N	*%*	mean (sd)	median	N	*%*	mean (sd)	median	χ ^2^/Z	P
**sociodemographics**															
total n		1037	*100*			693	*100*			344	*100*				
age (in years)				39.52 (12.56)	39.05			37.57 (11.97)	36.67			43.46 (12.80)	42.85	Z = -6.980	< 0.001*
education (in years)				11.94 (3.37)	12.00			11.71 (3.34)	12.00			12.42 (3.39)	12.00	Z = -2.714	0.007*
ethnicity:	caucasian	992	*95.66*			661	*95.38*			331	*96.20*				
	african	7	*0.68*			6	*0.87*			1	*0.30*				
	mixed	10	*0.96*			7	*1.01*			3	*0.90*			χ^2 ^= 1.202	0.753
	unknown	28	*2.70*			19	*2.74*			9	*2.60*				
native tongue:	German	902	*86.98*			591	*85.71*			311	*90.67*				
	bi-lingual German	46	*4.44*			38	*4.33*			8	*1.46*			χ^2 ^= 6.899	0.032*
	other	89	*8.58*			64	*9.96*			25	*7.87*				
marital status:	single	748	*72.13*			575	*82.97*			173	*50.44*				
	married	129	*12.44*			48	*6.93*			81	*23.32*				
	divorced	124	*11.96*			57	*8.23*			67	*19.53*			χ^2 ^= 121.516	< 0.001*
	widowed	13	*1.25*			3	*0.43*			10	*2.92*				
	unknown	23	*2.22*			10	*1.44*			13	*3.79*				
living situation:	alone	292	*28.16*			201	*29.00*			91	*26.45*				
	alone with children	17	*1.64*			0	*0*			17	*4.94*				
	with partner (± children)	137	*13.20*			50	*7.22*			87	*25.29*				
	With parents	157	*15.14*			121	*17.46*			36	*10.47*				
	with others (family members, friends)	71	*6.85*			53	*7.65*			18	*5.23*			χ^2 ^= 116.823	< 0.001*
	sheltered home	282	*27.19*			212	*30.59*			70	*20.35*				
	forensic hospital	54	*5.21*			43	*6.20*			11	*3.20*				
	homeless	4	*0.39*			4	*0.58*			0	*0*				
	unknown	23	*2.22*			9	*1.30*			14	*4.07*				
**clinical picture**															
diagnosis:	classical schizophrenias schizoaffective disorders	852 185	*82.16* *17.84*			615 78	*88.74* *11.26*			237 107	*68.90* *31.10*			χ^2^= 61.794	< 0.001*
age of onset of first psychotic episode				25.75 (8.81)	23.00			24.49 (7.71)	22.00			28.28 (10.23)	26.00	Z = -5.705	< 0.001*
duration of disease (in years)				13.23 (10.71)	10.87			12.57 (10.38)	10.16			14.54 (11.24)	13.02	Z = -2.600	0.009*
hospitalization (number of inpatient stays)				8.60 (9.76)	6.00			8.49 (9.95)	5.00			8.83 (9.38)	6.00	Z = -0.727	0.467
chlorpromazine equivalents				687.36 (696.85)	499.98			706.67 (668.43)	520.00			648.35 (750.50)	450.00	Z = -2.428	0. 015*
PANSS^a^:	positive symptoms			13.76 (6.32)	12.00			13.94 (6.16)	12.00			13.92 (6.64)	12.00	Z = -0.130	0.990
	negative symptoms			18.23 (7.85)	17.00			18.14 (7.57)	17.00			18.11 (8.44)	17.00	0.886	0.376
	general psychiatric symptoms			33.73 (11.83)	32.00			33.37 (11.31)	32.00			34.50 (12.81)	33.00	-0.886	0.376
	total score			65.64 (23.40)	63.00			65.32 (22.41)	63.00			66.31 (25.37)	62.00	-0.025	0.980
Clinical Global Impression scale^b^				5.57	6.00			5.57 (1.03)	6.00			5.57 (1.18)	6.00	Z = -0.121	0.894
Global Assessment of Functioning^c^				45.76 (0.68)	45.00			45.60 (16.30)	45.00			46.09 (19.11)	45.00	Z = -0.323	0.747
global quality of life ^d^				5.41 (2.37)	5.00			5.43 (2.31)	5.00			5.38 (2.49)	5.00	Z = -0.378	0.705
Brief Symptom Inventory ^e^:	general severity index			0.88 (0.68)	0.71			0.87 (0.66)	0.71			0.92 (0.72)	0.71	Z = -0.687	0.492
State-Trait-Anxiety Inventory ^f ^:	state anxiety			43.54 (10.89)	43.00			43.48 (10.45)	43.00			43.65 (11.79)	43.00	Z = -0.121	0.904
	trait anxiety			44.96 (11.34)	45.00			44.67 (11.09)	45.00			45.56 (11.82)	46.00	-0.983	0.326
Toronto Alexithymia Scale ^g^				2.59 (0.56)	2.61			2.58 (0.54)	2.55			2.60 (0.60)	2.66	Z = -0.607	0.544

^a^Kay SR, Fiszbein A, Opler LA: The positive and negative syndrome scale (PANSS) for schizophrenia. Schizophr Bull1987,13(2):261-276. ^b^Guy W: Clinical Global Impressions (CGI). In ECDEU Assessment manual for psychopharmacology, revised NationalInstitue of Mental Health. Rockville, MD; 1976. ^c^AmericanPsychiatricAssociation: Diagnostic and statistical manual of mental disorders, 4th edition (DSM-IV). Washington, DC: American Psychiatric Press; 1994. ^d^Based on a visual analogue scale (Krampe H, Bartels C, Victorson D, Enders CK, Beaumont J, Cella D, Ehrenreich H: The influence of personality factors on disease progression and health-related quality of life in people with ALS. Amyotroph Lateral Scler 2008, 9:99-107). ^e^Franke GH: Brief Symptom Inventory (BSI). Goettingen: Beltz; 2000. ^f^Laux L, Glanzmann P, Schaffner P, Spielberger CD: Das State-Trait-Angstinventar (STAI). Weinheim: Beltz; 1981. ^g^Kupfer J, Brosig B, Braehler E: Toronto Alexithymie-Skala-26 (TAS-26). Goettingen: Hogrefe; 2001.

### Traveling team

The GRAS team of traveling investigators consisted of 1 trained psychiatrist and neurologist, 3 psychologists and 4 medical doctors/last year medical students. All investigators had continuous training and calibration sessions to ensure the highest possible agreement on diagnoses and other judgments as well as a low interrater variability regarding the instruments applied. Patient contacts were usually prepared by colleagues/personnel in the respective collaborating psychiatric centers (Figure 2) to make the work of the travel team as efficient as possible.

### The GRAS manual

A standardized procedure for examination of the patients has been arranged with the GRAS manual, composed for the purpose of the GRAS data collection. Table 1 presents its contents, including established instruments, such as clinical interviews/ratings, questionnaires, cognitive and neurological tests [2,29-53].

### GRAS operating procedure

**Figure 4:**
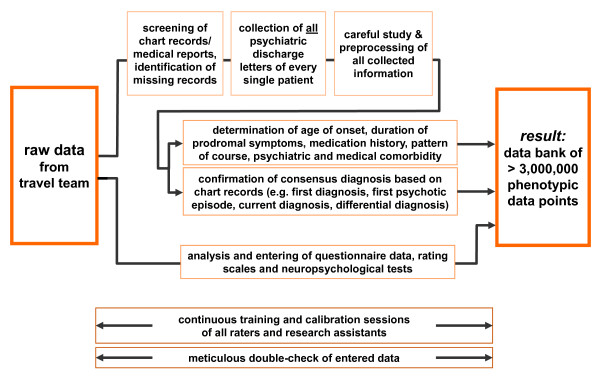
**Development of the GRAS data bank**. Raw data, brought to Göttingen by the traveling team of examiners, were only entered into the data base after careful and comprehensive data re-checking, also based on patient charts and discharge letters. During the whole process, continuous calibration sessions and repeated re-checking of the entered data took place.

**Figure 5:**
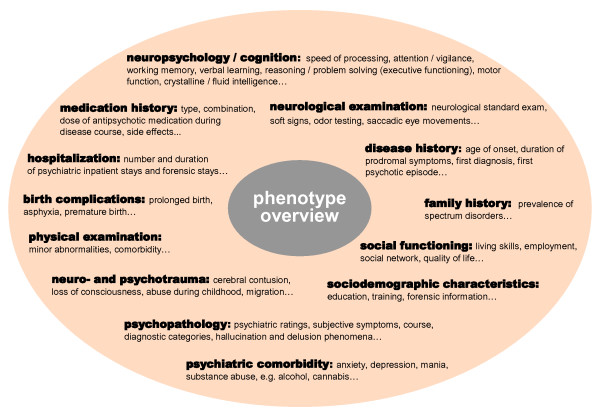
**Phenotype overview**. Various different domains covered by the GRAS data collection are displayed. These domains will also deliver the basis for further sophistication of phenotypical readouts.

The GRAS data base operating procedure leading from the large set of raw data provided by the travel team to the data bank with its several-fold controlled and verified data points is illustrated in Figure 4. Already during the time when the travel team examined patients all over Germany, a team of psychologists started to work on the development of the GRAS data base, integrating the raw data to ultimately result in over 3000 phenotypic data points per patient (total of over 3.000 000 data points at present in the data collection) (Figure 5). Most importantly, the chart records/medical reports of all patients were carefully screened, missing records identified and, in numerous, sometimes extensive and repeated, telephone and written conversations, missing psychiatric discharge letters of every single patient organized. After careful study and pre-processing of raw data and chart records, the confirmation of the diagnoses, determination of age of onset of the disease and prodrome as well as other essential readouts were achieved by meticulous consensus decisions.

### Statistical analyses

For the establishment of the data base and for basic statistical analyses of the data, SPSS for Windows version 17.0 [54] was used. Comparisons of men and women in terms of sociodemographic and clinical picture as well as neurological examination were assessed using either Mann-Whitney-U or Chi-square test. Prior to correlation and regression analyses, selected metric phenotypic variables were standardized by Blom transformation [55]. The Blom transformation is a probate transformation into ranks and the resulting standardized values are normally distributed with zero mean and variance one. Comparisons of men and women in terms of cognitive performance were assessed by analyses of covariance, using age, duration of disease, years of education and chlorpromazine equivalents as covariates. For all intercorrelation patterns, correlations of the particular target variables were assessed using Pearson product-moment correlation. Cronbach's alpha coefficient was determined for estimation of internal consistency of the target variables within a defined intercorrelation pattern. Multiple regression analyses using the enter method were conducted to evaluate the contribution of selected disease related variables (duration of disease, positive symptoms, negative symptoms, catatonic signs and chlorpromazine equivalents) to 3 dependent variables: basic cognition/fine motor functions, cognitive functions and global functioning (GAF) [2]. The dependent variables basic cognition/fine motor functions and cognitive functions are both composite score variables. The basic cognition/fine motor function score comprises alertness (TAP), dotting and tapping (Cronbach's alpha = .801) [39,46] and the cognition score consists of reasoning (LPS3), 2 processing speed measures (TMT-A and digit-symbol test, ZST), executive functions (TMT-B), working memory (BZT), verbal learning & memory (VLMT) and divided attention (TAP) [37,38,41,44-46] (Cronbach's alpha = .869). For both scores, a Cronbach's alpha >.80 indicates a high internal consistency as prerequisite for integrating several distinct items into one score. Multiple regression analyses were conducted for the total sample and separated for men and women.

## Results

### Biographic and clinical data

**Figure 6:**
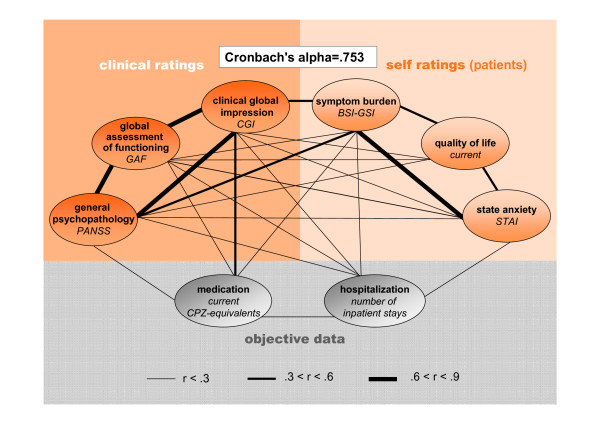
**Clinical intercorrelation pattern**. Correlations between measures of the clinical picture derived from different approaches: Patient self-ratings, clinical rater judgement and 'objective data'. Thickness of the lines represents the strength of correlation between two measures; only significant correlations are displayed. Note the strong internal consistency expressed by a Cronbach's alpha of .753.

The GRAS data collection comprises presently (as of August 2010) 1037 patients with confirmed diagnosis of schizophrenia (82.2%) or schizoaffective disorder (17.8%). A total of 693 men and 344 women fulfilled the respective diagnostic requirements of DSM-IV. Table 2 provides a sample description, both total and separated for male and female patients, with respect to sociodemographic data and clinical picture. There are some differences between genders in the GRAS sample: Women are older, less single, have more years of education, more diagnoses of schizoaffective disorders, longer duration of disease, later age of onset of first psychotic episode and lower doses of antipsychotics. However, regarding determinants of the clinical picture, e.g. PANSS scores [30], genders do not differ significantly. An intercorrelation pattern of selected clinical readouts, obtained by (1) clinical ratings and (2) self-ratings of the patients and complemented by (3) 'objective data', in this case medication and hospitalization, is presented in Figure 6. The Cronbach's alpha of .753 suggests that items derived from the 3 different perspectives harmonize well. Whereas patient ratings of quality of life and state anxiety (STAI) [32] are only weakly correlated with professional clinical ratings and objective data, the patients' self-estimated symptom burden as measured with the BSI [33] shows moderate to good correlation.

### Cognition

**Figure 7:**
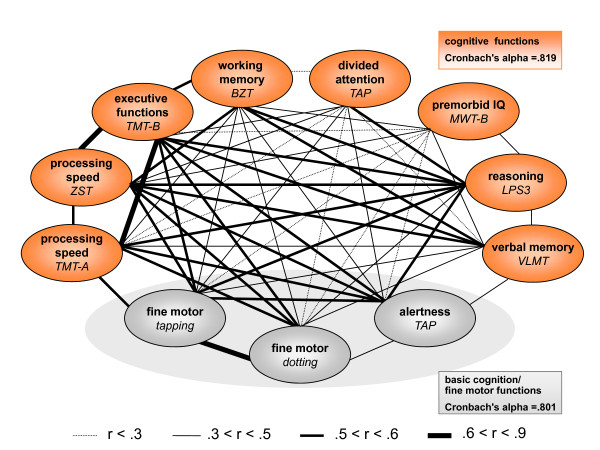
**Cognitive intercorrelation pattern**. Shown are all neuropsychological tests performed, together with their respective cognitive domain. Thickness of the lines represents the strength of correlation between two tests; only significant correlations are displayed. Tests for higher cognitive functions are labelled in orange; tests for basic (mainly basic cognition/fine motor dependent) functions in grey. Measures of higher cognitive functions as well as measures of basic cognition/fine motor functions show powerful internal consistency (Cronbach's alpha of .819 and .801 respectively).

For the ongoing/planned genetic analyses, not only the clinical picture with its schizophrenia-typical positive and negative symptoms, but particularly cognition plays an important role. The cognitive tests applied in the GRAS data collection show an intercorrelation pattern that further underlines quality and internal consistency of the data obtained by the invariable team of investigators (Figure 7). Table 3 represents the cognitive performance data of the complete GRAS sample in the respective domains. In addition, the performance level of men and women is given as well as - for comparison - available normative data of healthy individuals. Since for dotting and tapping [39], no normative data were available in the literature, the values shown in Table 3 were obtained from the healthy GRAS control population for cognitive measures (n = 103; see patients and methods).

**Table 3:** Cognitive performance of GRAS patients. For comparison, normative data are presented wherever available2.

	men			women			ANCOVA		total			normative data (PR) or mean sample values of healthy controls		
					
	N	mean (sd)	median	N	mean (sd)	median	F	p	N	mean (sd)	median	N	PR (Percentile Rank)	mean (sd)
**reasoning **(LPS)	663	21.26 (6.70)	22.00	324	18.79 (6.31)	18.00	17.62	< .001*	987	20.45 (6.67)	21.00	1556*^a^*	31	-
**working memory **(BZT)	627	13.24 (3.79)	14.00	312	12.62 (3.91)	13.00	1.20	.274	939	13.03 (3.84)	13.00	30*^b^*	-	15.70 (2.6)
**executive functions **(TMT-B)°	631	131.42 (104.21)	99.00	307	147.65 (121.09)	108.00	0.00	.956	938	136.73 (110.22)	100.00	24*^c^*	10	71.5 (31.07)
**verbal memory**^1) ^(VLMT)	602	41.15 (12.63)	41.00	302	42.68 (13.02)	42.00	12.38	< .001*	904	41.66 (12.78)	42.00	89*^d^*	10	52.39 (7.87)
**premorbid IQ**^1)^(MWT-B)	613	25.96 (6.22)	27.00	311	26.21 (6.13)	27.00	0.69	.405	924	26.04 (6.19)	27.00	1952*^e^*	43.5	-
**divided attention **(TAP)°														
*reaction time*	651	759.67 (114.25)	743.43	308	805.16 (150.99)	780.04	14.07	< .001*	959	774.28 (128.89)	755.05	200*^f^*	8	-
*lapses*		3.35 (7.15)	1.00		6.41 (13.18)	2.00	22.12	< .001*		4.33 (9.62)	1.00			
**processing speed**														
*trail making test A *(TMT-A)°	676	49.18 (35.22)	40.00	332	55.32 (42.22)	43.00	0.17	.683	1008	51.20 (37.76)	41.00	24*^c^*	< 5	33.04 (7.89)
*digit-symbol test *(ZST)	674	37.46 (12.58)	37.00	329	38.58 (14.14)	39.00	19.24	< .001*	1003	37.83 (13.12)	38.00	200*^g^*	16	-
**basic cognition/fine motor function**														
alertness (TAP)°														
*reaction time*	665	319.62 (116.13)	284.08	326	379.11 (161.80)	328.04	28.30	< .001*	991	339.19 (135.73)	298.41	200*^f^*	10	-
*lapses*		0.52 (2.04)	0.00		1.18 (3.57)	0.00	10.39	.001*		0.73 (2.66)	0.00			
*dotting*	673	46.10 (13.08)	46.00	320	45.36 (14.96)	46.00	1.62	.203	993	45.86 (13.71)	46.00	103*^h^*	-	63.24 (11.03)
*tapping*	671	29.01 (8.57)	29.00	319	27.58 (9.00)	27.00	0.76	.783	990	28.55 (8.73)	28.00	103*^h^*	-	37.63 (7.04)

° Higher scores reflect better performance, except for TMT-A, TMT-B, Alertness and Divided Attention (TAP)

* For statistical comparison (ANCOVA) between men and women values are corrected for age, duration of disease, chlorpromazine equivalents and years of education (except MWT-B).

^1) ^Non-native and non-bilingual German speaking patients are excluded (n = 89).

^2) ^Percentile ranks (PR) < 15 indicate that the mean or the median of the total sample is below average in comparison to a normative sample.

^a^Horn W: Leistungsprüfsystem (LPS). 2 edition. Goettingen: Hogrefe; 1983. ^b^Gold JM, Carpenter C, Randolph C, Goldberg TE, Weinberger DR: Auditory working memory and Wisconsin Card Sorting Test performance in schizophrenia. Arch Gen Psychiatry 1997, 54(2):159-165. ^c^Perianez JA, Rios-Lago M, Rodriguez-Sanchez JM, Adrover-Roig D, Sanchez-Cubillo I, Crespo-Facorro B, Quemada JI, Barcelo F: Trail Making Test in traumatic brain injury, schizophrenia, and normal ageing: sample comparisons and normative data. Arch Clin Neuropsychol 2007, 22(4):433-447. ^d^Helmstaedter C, Lendt M, Lux S: Verbaler Lern- und Merkfähigkeitstest (VLMT). Goettingen: Beltz; 2001. ^e^Lehrl S: Mehrfach-Wortschatz-Intelligenztest MWT-B. Balingen: Spitta Verlag; 1999. ^f^Zimmermann P, Fimm B: Testbatterie zur Aufmerksamkeitsprüfung (TAP). Version 1.02c. Herzogenrath: PSYTEST; 1993. ^g^Tewes U: Hamburg-Wechsler Intelligenztest fuer Erwachsene (HAWIE-R). Bern: Huber; 1991. ^h^Healthy controls recruited for selected cognitive and olfactory testing (unpublished data).

Comparing cognitive performance of schizophrenic men and women, analyses of covariance have been conducted, with age, duration of disease, years of education and chlorpromazine equivalents as covariates, which revealed significant gender differences in discrete cognitive domains. Men performed better in reasoning (F = 17.62, p <.001), alertness (F = 28.30, p <.001 for reaction time and F = 10.39, p = .001 for lapses), and divided attention (F = 14.07 p <.001 for reaction time and F = 22.12, p <.001 for lapses). In contrast, female schizophrenic patients were superior in verbal memory tasks (F = 12.38, p <.001) and digit symbol test (F = 19.24, p <.001). With respect to normative data obtained from healthy controls, cognitive data of all schizophrenic patients are in the lower normal range (percentile rank = 16 for digit symbol test) or even below (percentile ranks 10 for verbal memory, TMT-A, TMT-B, alertness and divided attention). Only for reasoning (LPS) [37] and premorbid intelligence (MWT-B) [36], schizophrenic subjects lie in the average range (percentile ranks of 31 and 43.5 respectively).

### Antipsychotic medication and side effects

**Figure 8:**
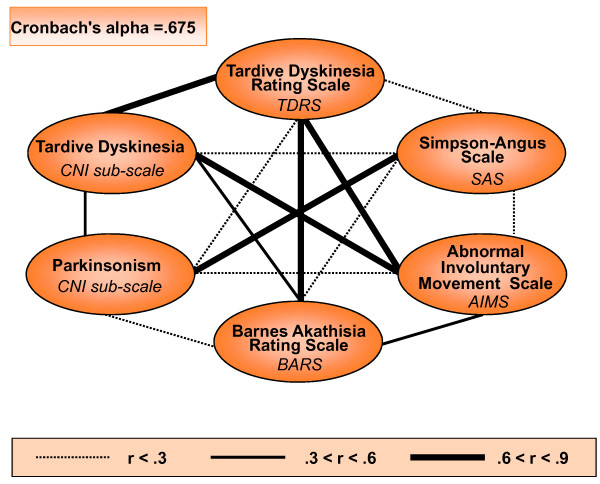
**Extrapyramidal intercorrelation pattern**. Shown are correlations between different neurological tests for measuring extrapyramidal symptoms. Thickness of the lines represents the strength of correlation between two tests; only significant correlations are displayed. Cronbach's alpha of .675 shows that these measures have a decent internal consistency.

Another important feature of schizophrenic patients that may influence their every-day functioning and performance, and result in a considerable number of side effects, is their antipsychotic medication. The GRAS data collection contains information on type, dose, duration of medication and drugs prescribed over the years. The mean dose of present antipsychotic medication of the whole GRAS population, expressed as chlorpromazine equivalents [56] amounts to 687.36 (± 696.85). Chlorpromazine equivalents in male are significantly higher as compared to female patients (Table 2). An overview of self-reported side effects of current antipsychotic medication in the GRAS sample, again sorted by gender, is given in Table 4. Of the 1037 patients with confirmed diagnosis of schizophrenia/schizoaffective disorder, 24 were presently not on antipsychotic drugs, whilst for 1 patient the current medication was unknown. Of the remaining 1012 patients who currently receive antipsychotic medication (16.5% first generation antipsychotics, 54.1% second generation antipsychotics and 29.4% mixed) and were all explicitly interviewed regarding medication side effects, only 423 reported any. The discrepancy between side effects measured versus side effects based on patients' reports becomes obvious when considering for instance the number of patients with clear extrapyramidal symptoms: A total of 335 subjects measured by Simpson-Angus Scale (mean score >.3) [50] contrasts only 117 patients self-reporting extrapyramidal complaints. External rating of extrapyramidal side effects in the GRAS population was comprehensively performed, utilizing a number of respective instruments which all showed significant intercorrelation (Figure 8) [47,49-52,57]. A composite score of the 6 Blom transformed scales, used for testing potential gender effects, yielded no significant differences in extrapyramidal symptoms in men versus women (Z = -0.022, p = 0.982).

**Table 4:** Self-reported medication side effects of patients (N = 423)* according to treatment type

	**FGA^1^**		**SGA^2^**	
		
	**men**	**women**	**men**	**women**
				
Parkinson symptoms	17%	15.6%	3.8%	11.6%
dyskinetic/dystonic symptoms	35.8%	31.3%	9.4%	9.7%
akathisia	22.6%	12.5%	6%	6.8%
hyperprolactinaemia	-	-	-	1.9%
hormonal dysfunctions (gynecomastia, absence/changes of menorrhea)	-	9.4%	-	5.8%
sexual dysfunction	7.5%	-	10.3%	-
vertigo (incl. hypotonia)	5.7%	12.5%	5.1%	8.7%
weight gain	9.4%	18.7%	38.3%	39.8%
diabetes mellitus	-	-	0.4%	-
sialorrhea ('drooling')	-	-	20.4%	6.8%
skin abnormalities, loss of hair	1.9%	-	1.7%	5.8%
gastrointestinal symptoms	1.9%	6.3%	5.9%	7.8%
hyperhidrosis	-	-	2.6%	-
psychological symptoms (loss of concentration, no drive, tiredness)	33.9%	28.1%	44.2%	31.1%
cardiovascular symptoms (tachycardia, hypertension)	-	-	1.3%	1.9%
impaired vision	-	-	1.7%	3.9%
dry mouth	5.7%	9.4%	5.1%	4.9%
urinary retention	-	3.1%	1.3%	-
number of patients who reported side effects	53	32	235	103

^1^FGA - first generation antipsychotics, typical antipsychotics

^2^SGA - second generation antipsychotics, atypical antipsychotics

*Only N = 423 patients (out of 1012 patients who were on antipsychotic medication) reported side effects (see text for details).

### Neurological symptoms

Similar to cognitive readouts, evaluation of inherent neurological symptoms in the schizophrenic patient population are of tremendous interest, not only for understanding the contribution of particular genes/genetic markers and/or environmental factors to the schizophrenic phenotype but also for estimating the impact of potential neurological comorbidities. Table 5 provides an overview of neurological symptoms based on the Cambridge Neurological Inventory (CNI) [47]. Only in the subscale 'Failure to suppress inappropriate response', significant differences between men and women (Z = -3.175, p = 0.001) became evident. Women were less able to hold respective responses back, e.g. to blink with one eye, leaving the other eye open, or to perform saccadic eye movements without moving the head.

**Table 5:** Cambridge Neurological Inventory (CNI)a subscale sum scores (N = 893-942)

	total		men		women		statistics	
				
sub scales	Mean (sd)	Median (range)	Mean (sd)	Median (range)	Mean (sd)	Median (range)	Z	p
*Hard neurological signs*								
plantar reflexes (le/ri*), power in upper and lower limb (le/ri), and reflexes (hyper- and hyporeflexia) in upper and lower limb (le/ri)	1.12 (1.70)	0.0 (0 - 10)	1.07 (1.66)	0.0 (0-8)	1.22 (1.78)	0.0 (0-10)	-1.467	n.s
*Motor coordination*								
finger-nose test (le/ri), finger-thumb tapping (le/ri), finger-thumb opposition (le/ri), pronation-supination (le/ri); fist-edge-palm test (le/ri), Oseretsky test	4.11 (4.27)	3.0 (0-20)	3.95 (4.17)	2.0 (0-20)	4.44 (4.45)	3.0 (0-20)	-1.629	n.s
*Sensory integration*								
extinction, finger agnosia (le/ri), stereoagnosia (le/ri), agraphesthesia (le/ri), left-right disorientation	3.66 (3.32)	3.0 (0-15)	3.63 (3.32)	3.0 (0-15)	3.73 (3.31)	3.0 (0-14)	-0.521	n.s
*Primitive reflexes*								
snout reflex, grasp reflex, palmo-mental reflex (le/ri)	0.84 (1.14)	0.0 (0-5)	0.80 (1.11)	0.0 (0-5)	0.91 (1.19)	0.0 (0-5)	-1.363	n.s
*Tardive dyskinesia*								
dyskinetic, sustained or manneristic face and head movement, simple or complex abnormal posture, dyskinetic, dystonic or manneristic trunk/limb movement	0.55 (1.17)	0.0 (0-9)	0.58 (1.25)	0.0 (0-9)	0.49 (0.98)	0.0 (0-7)	-0.132	n.s
*Catatonic signs*								
gait mannerism, gegenhalten, mitgehen, imposed posture, exaggerated or iterative movement, automatic obedience, echopraxia	0.43 (0.96)	0.0 (0-8)	0.45 (0.98)	0.0 (0-8)	0.38 (0.91)	0.0 (0-7)	-1.717	n.s
*Parkinsonism*								
increased tone in upper and lower limb (le/ri), decreased associated movements in walking, shuffling gait, arm dropping, tremor postural or resting, rigidity in neck	1.76 (2.90)	0.0 (0-15)	1.70 (2.85)	0.0 (0-15)	1.89 (3.02)	0.5 (0-15)	-1.172	n.s
*Failure to suppress inappropriate response*								
blinking or head movement in saccadic eye movement, winking with one eye	1.23 (1.49)	1.0 (0-6)	1.12 (1.42)	1.0 (0-6)	1.48 (1.62)	1.0 (0-6)	-3.175	.001*

*le/ri - left and right

^a^Chen EY, Shapleske J, Luque R, McKenna PJ, Hodges JR, Calloway SP, Hymas NF, Dening TR, Berrios GE: The Cambridge Neurological Inventory: a clinical instrument for assessment of soft neurological signs in psychiatric patients. Psychiatry Res 1995, 56(2):183-204.

### Prediction of functioning

In order to delineate the influence of disease on functioning in the GRAS sample, multiple regression analyses have been employed. These procedures assessed the contribution of 5 disease-related variables, i.e. duration of disease, PANSS positive and negative scores [30], catatonic signs [47], and dose of antipsychotic medication, to 3 dependent performance variables: (a) basic cognition/fine motor functions, (b) cognitive performance and (c) global functioning (Table 6). Regarding basic cognition/fine motor function, multiple regression analysis revealed a significant model accounting for 32.4% of variance in the total sample. In fact, duration of disease, negative symptoms, catatonic signs, and medication (chlorpromazine equivalents) contributed significantly to basic cognition/fine motor function, whereas positive symptoms did not (β = -.006, p = .856). According to the standardized regression coefficients, duration of disease and negative symptoms are the best predictors of basic cognition/fine motor function (β = -.346, p < .001 and β = -.334, p < .001). For higher cognitive functions, the set of disease-related variables explained 33% of variance in the total sample. Again, duration of disease and negative symptoms are the best predictors of higher cognitive functions (β = -.335, p < .001 and β = -.351, p < .001). Positive symptoms did not reach significance (β = - .015, p = .658). With respect to global functioning, all chosen disease-related factors accounted for 59.6% of variance in the total sample. Only duration of disease *per se *did not reach significance (β = -.028, p = .198). Positive and negative symptoms were the strongest predictors of global functioning (β = - .441, p < .001 and β = -.380, p < .001).

**Table 6:** Multiple regression analyses predicting a) basic cognition/fine motor functions, b) cognitive performance, c) global functioning

	total			male			female		
			
	β	t	p	β	t	p	β	t	p
**a) basic cognition/fine motor functions^1^**									
duration of disease (*years*)	-.346	-11.92	< .001	-.353	-9.68	< .001	-.318	-6.59	< .001
positive symptoms (*PANSS*)	-.006	-0.18	.856	-.028	-0.69	.489	.065	1.08	.283
negative symptoms (*PANSS*)	-.334	-10.05	< .001	-.293	-7.32	< .001	-.415	-7.01	< .001
catatonic signs (*CNI*)	-.126	-4.26	< .001	-.128	-3.45	.001	-.161	-3.27	.001
medication (*CPZ-equivalents*)	-.080	-2.70	.007	-.066	-1.83	.068	-.147	-2.84	.005
***regression model***		*r^2 ^= .324* *p < .001*			*r^2 ^= .306* *p < .001*			*r^2 ^= .383* *p < .001*	
**b) cognitive performance^2^**									
duration of disease (*years*)	-.335	-11.54	< .001	-.356	-9.72	< .001	-.294	-6.12	< .001
positive symptoms (*PANSS*)	-.015	-0.44	.658	-.033	-0.80	.427	.023	0.38	.704
negative symptoms (*PANSS*)	-.351	-10.47	< .001	-.320	-7.92	< .001	-.396	-6.56	< .001
catatonic signs (*CNI*)	-.132	-4.46	< .001	-.103	-2.76	.006	-.204	-4.16	< .001
medication (*CPZ-equivalents*)	-.082	-2.74	.006	-.060	-1.62	.105	-.140	-2.70	.007
***regression model***		*r^2 ^= .330* *p < .001*			*r^2 ^= .305* *p < .001*			*r^2 ^= .394* *p < .001*	
**c) global functioning^3^**									
duration of disease (*years*)	-.028	-1.29	.198	-.008	-0.28	.780	-.062	-1.78	.076
positive symptoms (*PANSS*)	-.441	-17.33	< .001	-.458	-14.45	< .001	-.415	-9.60	< .001
negative symptoms (*PANSS*)	-.380	-15.02	< .001	-.345	-10.97	< .001	-.430	-10.0	< .001
catatonic signs (*CNI*)	-.060	-2.67	.008	-.050	-1.71	.088	-.093	-2.58	.011
medication (*CPZ-equivalents*)	-.106	-4.71	< .001	-.122	-4.29	< .001	-.078	-2.07	.040
***regression model***		*r^2 ^= .596* *p < .001*			*r^2 ^= .559* *p < .001*			*r^2 ^= .662* *p < .001*	

^1^A basic cognition/fine motor composite score was used as a dependent variable comprising alertness (TAP), tapping, and dotting (Chronbachs alpha = .801).

^2^A cognitive composite score was used as a dependent variable consisting of reasoning (LPS3), 2 processing speed measures (TMT -A and digit-symbol test, ZST), executive functions (TMT-B), working memory (BZT), verbal memory (VLMT) and divided attention (TAP) (Chronbachs alpha = .869).

^3^Global assessment of functioning (GAF) was used as a dependent variable.

## Discussion

The present paper provides an overview of the GRAS data collection, including (1) study logistics and procedures, (2) sample description regarding sociodemographic data, disease-related variables, cognitive performance and neurological symptoms, paying particular attention to gender differences, and (3) a first presentation of intercorrelation patterns for selected areas of interest to phenotype studies. (4) In addition, disease-related factors influencing important criteria of daily functioning are evaluated in the >1000 GRAS patients. Overall, the GRAS sample represents a typical schizophrenic population in contact with the health system and is - last not least due to its homogeneous data acquisition - ideally suited for the ongoing and planned phenotype-based genetic association studies (PGAS) (e.g. [[11], and Grube et al: Calcium-activated potassium channels as regulators of cognitive performance in schizophrenia, submitted]).

The GRAS data collection has several remarkable advantages, two of which are of major importance for its ultimate goal, PGAS: (i) Different from other studies dealing with the establishment of a schizophrenia data base, *all *data for GRAS were collected by one and the same traveling team of examiners, who frequently performed calibrating sessions and rater trainings. This effort has clearly paid off in terms of reliability and quality of the data, considering the internal consistencies of the GRAS phenotypes, as exemplified in the displayed correlation patterns. (ii) Even though the GRAS study has been implemented as a cross-sectional investigation, the GRAS data collection also includes solid longitudinal information derived from the almost complete psychiatric chart records/discharge letters of all schizophrenic patients. This longitudinal set of data has been essential to e.g. reliably estimate prodrome versus disease onset, i.e. occurrence of the first psychotic episode.

Comparable to other schizophrenia samples, the GRAS sample comprises around two thirds of male and one third of female patients [17,58]. Assuming that the gender ratio in schizophrenia were 1:1 as claimed in text books, but recently also questioned [59,60], then two principal reasons may account for the gender distribution observed here: (1) Schizophrenic women generally seem to have less contact with the health system due to being better socially settled (later age of onset of disease) and protected within their families [61]; (2) A certain (smaller) recruitment bias may be explained by the fact that the traveling team of examiners visited some institutions with an overrepresentation of males, e.g. specialized forensic units or a hospital for psychotic patients with co-morbid substance use disorders.

With the purposeful strategy to visit several different facilities of psychiatric health care covering inpatients, outpatients, residents of sheltered homes and forensic patients, the GRAS approach tried to avoid biases inherent to pure inpatient samples [58]. Nevertheless, patients who are not in contact with the health care system are unlikely to be integrated in any comparable data bases. For instance, only 4 of the 1085 examined patients are currently homeless, whereas among homeless people a considerable proportion suffers from schizophrenia [62]. To reach them as well, different and more cost intensive recruitment strategies would be required [13]. On the other hand, the schizophrenic phenotype required for the GRAS-PGAS studies pursued here, might be veiled in this severely affected subsample of patients that is additionally characterized by other specific problems, e.g. a highly elevated incidence of multiple substance use disorders and severe downstream medical comorbidities [63,64].

Gender differences in schizophrenia as obvious from the present data collection have been known for a long time [65]. In agreement with the literature, men and women in the GRAS sample differ by diagnosis, with women having a higher rate of schizoaffective disorders [66,67]. With respect to age of onset, education, indicators of social integration (e.g. marital status, living situation) and medication, the present results are also in perfect agreement with previous findings: Male patients are younger when the first psychotic episode occurs, are more frequently single, more often dependent on supported living conditions (e.g. residential homes) and show lower educational status [61,67,68]. Among the explanations for the observed gender differences in schizophrenia are the protective role of female hormones [69] and social aspects like earlier marriage of young women leading to a more protected environment at disease onset [13]. In line with these considerations is the work of Häfner and colleagues [12]. In a prospective design he could show that 'the social course (of schizophrenia) is determined by individual stage at illness onset and by early illness course' [70].

With respect to psychopathology and premorbid functioning, the GRAS sample may be slightly different from other schizophrenia samples reported in the literature [67]. Several studies published in this area show that men exhibit more negative symptoms, even in a geriatric sample [71,72], and that females have poorer premorbid cognitive functioning than males [73]. In the GRAS patients, there are no gender differences regarding psychopathology and premorbid cognition. Importantly, clear support for a comparable severity of psychopathology in men and women of the GRAS sample is provided by the lack of gender differences in numbers of hospitalizations, clinical severity ratings, including global functioning (CGI, GAF [2,31]), and self-ratings of symptom severity and anxiety. One potential explanation for the discrepancies between the GRAS sample and other studies regarding psychopathology may be that patient numbers in some of the other studies have been too low to give conclusive results. In the assessment of premorbid cognitive functioning of the GRAS sample, a methodological limitation could be the retrospective determination of premorbid intelligence using a so-called 'hold' measure, i.e. a multiple choice vocabulary test [35]. Even though this is an accepted and valid instrument to retrospectively estimate premorbid intelligence [74], a prospective procedure might be more accurate. In fact, Weiser and colleagues had the opportunity to base their assessments on cognitive testing performed on adolescents before starting their military service [73], potentially explaining the deviating results.

Gender differences regarding current cognitive performance are similar within the GRAS sample (even though at a lower functioning level [75]) compared to healthy controls [76] after considering age of onset, duration of disease, education and medication as covariates. Men perform better in reasoning, alertness and divided attention but worse in verbal memory, confirming reports on first-episode as well as chronically ill schizophrenic patients [77].

Women in the GRAS study receive significantly lower doses of chlorpromazine equivalents, confirming that they require less medication to achieve a reasonable treatment effect [78]. Importantly, regarding medication side effects, there were no gender differences in extrapyramidal symptoms. There were also no differences in the overall proportion of men and women who self-reported side effects, but the pattern of complaints was slightly different. For instance, women mentioned more often hormonal dysfunction and vertigo (or related symptoms like hypotonia), whilst men complained mainly about sexual dysfunction. Altogether, it is worth pointing out that the percentage of patients self-reporting side effects is low when compared to that with objectively measured side effects, e.g. extrapyramidal symptoms (11.3% versus 32.3%).

Explicit studies on gender differences in antipsychotic medication side effects found a somewhat different distribution of complaints, e.g. more weight gain, diabetes and specific cardiovascular diseases in females [78,79]. Here, one reason is certainly the still preliminary data set of the GRAS collection evaluated, based at this point exclusively on cross-sectional patient reports. For a more appropriate coverage of medication side effects, all charts/discharge letters of every GRAS patient (also of those patients who did/could not report them), will have to be screened and entered into the data base. Comprehensive information on antipsychotic (and other) drugs and their side effects in the GRAS sample has been collected and is waiting for analyses to support e.g. future pharmacogenomic approaches, perhaps also in collaboration with industry partners.

In line with the findings of a recent meta-analysis [80], positive symptoms of the GRAS patients do not influence higher cognitive function or basic cognition/fine motor performance, whilst negative symptoms, catatonic signs, duration of disease and antipsychotic medication have a significant effect on both. The clinical ratings of global functioning, however, strongly rely on positive as well as negative symptoms, medication and catatonic signs [81-83].

## Conclusion

GRAS enables a novel phenotype-based approach to understand the molecular-genetic architecture of schizophrenia. The GRAS data collection encompasses a large sample of comprehensively phenotyped, moderately to severely affected schizophrenic patients. Proof-of-principle for the suitability of the GRAS data collection for PGAS has already been demonstrated [[11], and Grube et al: Calcium-activated potassium channels as regulators of cognitive performance in schizophrenia, submitted]. Further extensive analyses of the accumulated information on every single patient are ongoing.

## Abbreviations

GRAS: Göttingen Research Association for Schizophrenia; GWAS: genome-wide association study; PGAS: phenotype-based genetic association study; CATIE: clinical antipsychotic trials of intervention effectiveness; CNI: Cambridge Neurological Inventory; ASRB: Australian Schizophrenia Research Bank; FGA: first generation antipsychotics; SGA: second generation antipsychotics; CPZ: chlorpromazine.

## Competing interests

The authors declare that they have no competing interests.

## Authors' contributions

MB coordinated and supervised the traveling team of investigators and had a considerable impact on design and establishment of the data collection. KR and HFr were part of the traveling team of investigators, conducted statistical analyses of the clinical data, assisted in manuscript writing, and supervised data entry, substantially performed by SG, SP, AK, MFG, VA, ATa, ATr, and MF. Of the collaborating centers, LA, JBA, MBE, TB, AC, MD, HFo, RF, RG, SH, DH, GK, HK, MFr, FL, WM, AM, RMI, CO, FGP, TP, US, HJS and UHR enabled the work of the traveling team of examiners, by pre-selecting and preparing patients and organizing respective facilities and working conditions. HE, KAN, NB, PF, WS, and JF developed the concept of GRAS (Göttingen Research Association for Schizophrenia, founded in 2004), and guided the project, data analysis, and paper writing, hereby supported by BKH. All authors discussed the results, commented on the paper draft and approved the final version of the manuscript.

## Pre-publication history

The pre-publication history for this paper can be accessed here:

http://www.biomedcentral.com/1471-244X/10/91/prepub
